# Syphilis, the Importance of a Thorouh Diagnosis and Long-Continued Treatment

**Published:** 1885-11

**Authors:** J. C. Le Hardy

**Affiliations:** Savannah, Ga.


THE ATLANTA MEDICAL AND SURGICAL JOURNAL.

VOL. II. NOVEMBER, 1885. NO. 9.

### Original Communications.

### SYPHILIS—THE IMPORTANCE OF A THOROUGH DIAGNOSIS AND LONG-CONTINUED TREATMENT.

READ BY TITLE AT THE 36TH ANNUAL SESSION OF THE MEDICAL ASSOCIATION OF GEORGIA, AT SAVANNAH, GA., APRIL 15TH, 1885, BY J. C. LE HARDY, M. D., SAVANNAH, GA.

In a paper, entitled "The Duality of the Syphilitic Poison," read before this Association in 1874, I briefly called the attention of the members to the pathological differences between syphilitic chancre and venereal ulcer, unwisely called by some authors "soft chancre" and "chancroids." Cases in illustration were cited, showing that the first is a constitutional disease having two different stages, one seemingly local (the chancre), the other general (secondary eruptions, etc.); that it is transmissible to posterity, and that mercury is the most potent remedy we possess for its cure; while the second is a purely local disease, having but one stage, not hereditary, and mercury is useless and often injurious in its treatment. I urged upon the profession the necessity of making an early diagnosis between these two diseases, in order to avoid the undiscriminating use of mercury, an error into which physicians have fallen, under the impression that they both originated from the same poison.

The additional experience of eleven years in the daily treatment of syphilis has demonstrated to me still more fully the necessity for a thorough investigation and careful study of every case of suspicious ulcer before making a diagnosis.

The exact nature of the disease should be so firmly *fixed* in the mind of the attending physician, that no after circumstance, not even the disappearance of all the symptoms, in a case diagnosed as syphilis, should shake his conviction or cause him to relax his efforts during the whole period necessary for the cure of that disease. For I am fully convinced that most of the cases of uncured spyhilis, with their dreadful consequences, are the direct result of an insufficient treatment. The chancre being healed and no secondary eruption appearing, the treatment is not persisted in, and the patient is allowed to marry whilst the poison is still lurking in his blood, to plague him in the future, or to infect his wife and curse his children with the hereditary form of the disease, the most disastrous perhaps of all in its effects upon humanity. This insufficiency may be due to the patients leaving the treatment as soon as possible, in order to save expense, or to the fault of the attending physician, who, through neglect, apathy or ignorance, will not rigidly enforce the necessarily long and wearisome treatment; or, again, it may be that through a lack of confidence in his own diagnosis, he allows his patient to discontinue a treatment which he does not deem necessary.

A patient will often present himself to the busy practitioner, with a sore upon the penis, having the characteristics of a chancre. Upon inquiry, the doctor ascertains that it made its appearance ten or twelve days after coition. He concludes that it is a syphilitic chancre, and without further examination treats it as such. A course of mercury is commenced, together with local treatment. In two or three weeks the sore heals perfectly. The mercurial treatment is kept up regularly during four or five months, when, no secondary symptoms having occurred, the patient being to all appearance perfectly healthy, the doctor concludes that he must have made a mistake, that the disease is not constitutional, and, yielding to the patient's importunities, discharges him as cured. In coming to this conclusion he may have committed a fatal error. Had he, in the first instance, made a careful study of the sore, and found under the microscope the syphilitic microbe, before and after cultivation, or had he inoculated the patient with its virus, without reproducing its like; had he made a thorough investigation of the surrounding parts and found the inguinal ganglia all enlarged, hard and painless on pressure, he would have been convinced that the case was one of syphilis, and never would have relaxed in his treatment for at least two years, and not then, unless the inguinal glands had returned to their normal condition, and all other symptoms had disappeared.

With the universal dread of syphilis existing, at the present day, among civilized people, due in a great measure to the pamphlets and other flaring advertisements of charlatans and patent medicine venders, any sore or abrasion upon the genital organs is regarded with fear and apprehension, and doctors are constantly called upon to give an opinion in cases where, if any disease exist at all, the symptoms are not sufficiently developed to declare its true character. Many physicians, still believing that the early use of caustics will destroy the virus and prevent the development of syphilis, or, yielding to the importunities of the patient, who wants something to be done at once, or perhaps to *the fear of losing a case*, sear the parts with these agents to such an extent as to mask the true nature and destroy the diagnostic value of the lesions which may appear later.

Applications of caustics at this period therefore render the diagnosis mere guess work and the treatment necessarily becomes hap-hazard. The effect of caustics upon a simple abrasion, existing upon the penis, may be to produce an ulcer with infiltration of the sub-tissues, liable to be mistaken for a syphilitic chancre, or it may produce an ulcer, causing a painful inflammation of the inguinal glands, with or without suppuration, thus similating a venereal ulcer. The patient may then be subjected for years to a course of mercurials for the cure of syphilis when none exist; and again, he may be treated for venereal ulcer, without mercury, and told that he has no constitutional disease, when really he is infected with syphilis—the symptoms of this disease having been hidden by the use of caustics. Thus he is exposed to the risk of general syphilis and its consequences, and the doctor, who makes a diagnosis without a sure foundation, suffers mortification and well-merited loss of reputation, when such a patient turns up with secondary eruptions.

The conscientious physician will not commence the treatment of any disease until he has made a satisfactory diagnosis, and it is especially important that this rule shall be strictly followed where such a serious malady is suspected.

In every suspicious case, the subject should be kept under daily observation, using such local applications only as will not interfere with the natural development of the disease. While waiting for this development, the doctor should make his patient fully understand the necessity for a long-continued treatment of the case in the event it should prove to be syphilis. It is of the greatest importance that this fact should be thoroughly impressed upon his mind, because the success of the treatment depends entirely upon its regularity and long continuance. Subjected early to treatment, the primary symptoms of syphilis are usually mild; when mercury is used from the inception, the secondary manifestations may or may not present themselves at the ordinary period, or never at all. When they do appear, it is generally in the form of superficial eruptions, which fade away in a short time, leaving the health of the patient seemingly unimpaired; yet, slight as these symptoms may be, unless a regular treatment be kept up, they will in most instances be followed, at some future period, by other and more serious exhibitions, which will undermine the constitution of the strongest subject, and carry his wreck to the grave. These are the cases which, to this day, lead many of us to believe that syphilis is not only incurable in this world, but that it will follow our shades into the next.

The large percentage of mild cases occurring under the mercurial treatment now in vogue, and the contrast between their symptoms and those of the same disease, as described by old authors, have led many prominent physicians and writers, both at home and abroad, to the opinion that syphilis is dying out by "evolution," or by "dilution." They believe that the germ is gradually becoming weaker and less dangerous as it passes through successive generations. Herbert Spencer, in his "Study of Sociology," seems to lend the weight of his great name to strengthen this false opinion; but it must be remembered that his object was to fight against the passage (by the English Parliament) of an act which he believed aimed at the exercise and consequent abuse of irresponsible power, and the testimony which he brings up to sustain the hypothesis that syphilis is diminishing in intensity and frequency in England is naturally *ex parte.*

An experience of thirty years, devoted in great part to the study and treatment of syphilis, has taught me that it is to be found in all conditions of society, among the pure and the licentious, the young and the aged, the married and the single. Sometimes, in its primitive form, with all the nauseous symptoms described by early writers; sometimes in a modified or mild form; sometimes so masked that its presence is not suspected until the disease for which the patient is under treatment, failing to yield to other measures, mercury is resorted to, when the benefit resulting brings the attention to syphilitic symptoms not before noticed.

That it now exists in its most malignant form in many parts of the world cannot be disputed. Our worthy President, Dr. Foster, proves this beyond a doubt in his essay, "Syphilis as a Sociological Problem," read before this Association last year, by copious extracts from the writings of men of recognized authority. My own observation also goes to prove that malignant syphilis, with the same symptoms of nauseating sores, phagædena, gummous tumors, disfiguring skin eruptions, and loss of hair, the excruciating night pains, the nodes and the various nervous affections described nearly four hundred years ago, is still to be met with in this country, especially when the disease is badly treated, or not treated at all, as is often the case among backwoodsmen, sailors and unsuspecting married women, and again under certain peculiar conditions of health, even when the most careful treatment is pursued.

That the mild form in which the disease is usually observed is the result of treatment is aptly illustrated by its history among the colored population of the Southern States. When the negroes were slaves they were well clothed, well fed and well housed; they were kept on the plantation under strict hygienic regulations; they were made to observe the marriage relations; when they became sick they were treated at once by competent physicians. Almost every Southern doctor knows *how rare were the cases of syphilis among the slaves.* When, however, through the result of the war, these people were set at liberty, a different state of things commenced. Large numbers left the plantation to follow the army. Many of the women contracted syphilis from the soldiers. Naturally careless and lazy, ignorant of all sanitary laws, perfectly demoralized, they huddled together in filthy, unventilated shanties and huts. Unrestrained in their sexual intercourse the disease became epidemic among them. Few escaped it in some phase or other, and the malignant form was the most predominant, and proved very fatal. Deprived of the fostering care of the master, ill fed, badly clothed, with little or no medical attention, the mortality was very great; much of it was due to syphilis, although not often attributed to it. When the disease attacked the lungs, the brain, or other vital organ with fatal results, the cases were recorded in mortuary reports, if recorded at all, under the name of consumption, paralysis, convulsions, etc. While this epidemic existed among the negroes, syphilitic cases also increased in number, in the surrounding white population, but it is to be noted that although the virulence of the primary sores was greater than usual, and the secondary manifestations more frequent and persistent, still under strict and regular treatment the disease was cured in the ordinary time.

To the sociologist and statesman a fact of importance to be observed in relation to this disease is, that, whether in its mild or in its aggravated form, syphilis is equally dangerous, if we consider its influence by heredity upon the constitution and the health of the masses, a subject which is beginning to attract the attention of medical investigators. From this point of view, perhaps the milder form is the most dangerous of the two, because a person with malignant syphilis is personally disagreeable; he shuns the society of others, and being avoided by every one is not liable to propagate the disease through contact; moreover, a severe case is likely to be treated with all the necessary care long enough to eradicate the poison from the system; while the milder form, on account of its very mildness, is liable to be neglected and remain uncured. Unrestrained in his social intercourse, such a person can readily transmit it through the lips, in the act of kissing (as he may have mucous patches in his mouth or throat, unknown to himself the most frequent mode of transmission in the secondary form among young people), by contact with objects used in common (among cigar-makers, glass-blowers, etc.,) or, if he marry, he may infect his wife by actual intercourse, or through the fœtus; and, again, his child, if born alive, may become a center of infection.

Since this disease, then, is not confined to the vicious and the depraved, but may affect virtuous women and innocent children as well; since it does not depend upon licentious and debauched habits for its propagation, but may be spread by the most innocent social intercourse, it is evident that all attempts to prevent its ravages by means of laws to license and control prostitutes must necessarily prove futile. It has passed beyond the reach of the law, if indeed history does not prove that it never was within its reach. So, then, I agree with Herbert Spencer in his opposition to such laws, but it seems astonishing, indeed, that so clear and deep a thinker should have supported this opposition in any part by *an argument attempted to be drawn from a comparison of the mortality from syphilis and from other diseases.* Such an argument is unphilosophical and vicious in its tendencies; it belittles the importance of the disease as an element in the problems of sociology, and blinds those who accept his reasoning to the vastness of the evil. To make it sound, we must be able to say in every case that death was or was not the result of syphilis, either directly or indirectly, and that is impossible.

Like malaria, but in a different manner, syphilis poisons the system, undermines the constitution, saps the vital strength, and diminishes the power to resist the attacks of other diseases. It complicates or masks the symptoms of other diseases. It impresses upon them its own refractory and malignant character, and thus many deaths, attributed to other causes, ought to be charged to syphilis, *but are not, if it can possibly be avoided.* (In the mortuary records of this city may be found the names of hundreds of persons reported as having died of consumption, softening of the brain, locomotor ataxia, convulsions, paralysis, scrofula, rheumatism, etc., etc., who, to my knowledge, were the subjects of general or of inherited syphilis.)

It may even be that in the process of "evolution," so much prated about at the present day, many diseases which now baffle the skill of the physician are the direct product of the syphilitic microbe. If such be the case, the dilution of the poison, like the homœopathic dilution, must increase its potency the further it is carried, if we consider the numbers who die from these diseases.

But this question, like all others pertaining to the operations of nature, can only be settled by the close and laborious study of an immense number of recorded facts, collected for years from a wide and varied field, and to this work it should be the pride of every physician to contribute his share.

Let the practitioner consult his records of past years, let him fathom the recess of his memory, and tell to the world how many of his patients have left treatment before they were cured ; how many discharged as cured (stating length of treatment) have returned with the secondary or tertiary form of the disease ; how many have called upon him to attend their wives for syphilis or abortions induced by it; how often, after the birth of a child, he has found it syphilitic. Let him give his experience of mothers infected by their own offsprings, of nurses, or friends infected by the nursling. Let him also recount those unaccountable complications which have appeared in the ailments of those who had been previously infected with syphilis, and the proportion of cases of consumption, scrofula, softening of the brain, paralysis, ataxia, etc., which present a syphilitic history. Let such similar information be gathered from the records of hospitals, civil, military and naval, public and private. In the mass of fact thus collected materials will be found for the building up of the true theory of the pathology of syphilis, and when the investigations, now in progress, into the natural history of the syphilitic microbe, shall have given us a correct knowledge of its growth and mode of propagation, the means of destroying this germ will, I doubt not, also be discovered, and we shall, without the aid of the law, have full control of the disease before it can become constitutional and hereditary. Thus our children will be protected against the infernal taint which now permeates all civilized society.

But that part of the subject is beyond the scope of this essay; my object is simply to give our Southern country doctors, who have but rare opportunities of seeing cases of syphilis and of studying the disease, a few practical directions which will, I hope, enable them to recognize it when they see it, and guard their communities against its spread.

### DIAGNOSIS.

Even to one who is in the daily habit of seeing the disease, the diagnosis of some cases of chancre is very difficult to make; hence it is not to be wondered at that mistakes are frequently made by the general practitioner. Thus it often occurs that cases come to me in which pimples, insect bites, herpes, venereal ulcers, warts, gonorrhœal infiltration of the prepuce, etc., had been cauterized and then treated as chancres. On the other hand, I often see patients with syphilis in its various forms, who had been treated for other complaints, as in this case now under treatment: A negro woman, with a chancre involving the pharynx, was treated for ulcerated sore throat, getting worse every day; she consulted another doctor, who, finding the woman's health broken down, and the glands of the neck much enlarged, treated her for scrofula, with iron tonics, continuing to cauterize the throat. Not improving under this treatment, she was brought to me by her employer, whose children she was nursing all the time!

I found, on examination, a large, angry ulcer in the throat, with a peculiar grayish slough at the bottom, all the lymphatic ganglia on the scalp, neck, axillæ and groins enlarged, hard, movable and painless. There was also a well-defined specific eruption between the shoulders, on the back and chest (a papular syphiloderm), one mucous patch on the pharynx, a fissure at one corner of the mouth, and three patches at the verge of the anus. In addition to these symptoms, the temperature was 97°, the skin mealy, and she looked very badly. With these unmistakable signs before me, I pronounced the case one of general syphilis, and treated her for it with favorable results so far.

In the investigation of any suspicious sore or erosion upon the genital organs of man or woman, or, indeed, upon any part of the body, let me advise you to be always guarded in the expression of an opinion. Take ample time to complete your investigation, and do not commit yourself until you are fully satisfied of its nature. Should any caustic application have been made, use a water-dressing or other placebo, and allow all of the effects of the cauterization to pass away; then wait upon the development of the ulcer. Never tell a patient, who has had an ulcer or abrasion or fissure upon the genitals, that he has not syphilis until after the expiration of several weeks, and not then, if you find the inguinal glands enlarged.

The chancre is always the result of an inoculation with the virus from a chancre, or from some of the secondary manifestations of syphilis, or from the blood of a person suffering with general syphilis. It is usually conveyed from man to woman, or woman to man, in the act of coitus; but may be in kissing, by the saliva, or by actual contact with a sore. It may be propagated from the nipple of the infected, from the fœtus in utero and the new-born babe. The knife or other instruments of the surgeon, the finger of the gynecologist, or of the accoucheur, may all be the vehicle for conveying the virus. Hence, the doctor must not conclude that his patient has no syphilitic disease because there is no evidence of his ever having had a primary sore on the genitals or an impure coition.

By far the most common site of inoculatiou is on the glans, or on the prepuce near the corona glandis, above or below; at the fourchette or the introitus vaginæ; but I have seen it on every part of the penis and its appendages, on the labia and mons veneris, in the vagina, the uterine neck, inside and outside, the urethra, the anus, in the rectum, on the toes, the legs, the thigh, the groins, the fingers, the hand, the nose, the lips, the tongue, the throat, the eyelid, the forehead, etc. Therefore, the search for a primary lesion should extend over the whole body, mucous membrane and skin alike.

The true chancre is almost invariably single; if ever multiple, all arise from one inoculation and appear at or nearly at the same time, in contrast with the venereal ulcer, which reproduces itself by new inoculations, and appears on successive days.

Its characteristic beginning is that of an elevated papule, which, after two or three days, becomes cup-shaped, from the dying and shrinking of the tissues at the centre. It may, however, first appear as a simple erosion or fissure, or a superficial ulcer, which may heal without leaving any cicatrix or induration behind, in which case, of course, they have no diagnostic value of their own, but when followed by an induration of the tissues beneath this fact is of the greatest importance.

The papule is usually small and flattened, but the resulting chancre may grow to the size of a ten cents piece. A chancre very rarely suppurates unless irritated or inflamed by some artificial cause.

Its contents are the disintegrating tissues, grayish in color, elastic, are tough, with a tendency to separate from the living tissues. At the line of demarkation there is an oozing of serum, which spreads over the surface of the ulcer and keeps it moist and shiny.

It is painless and heals rapidly as soon as the dead tissues are detached. If not irritated by treatment, it will be entirely covered with epithelium in one to three weeks, leaving a persistent discoloration above and an induration beneath, which may last for months. When situated on a mucous membrane, the papule and the resulting chancre are flat; the induration beneath is thin and parchment-like.

The erosion or fissure may involve only the epithelium and underlying cellular tissue; if the specific induration follows, it usually extends into the cutis vera.

The chancre, beginning as a papule on the skin, penetrates into the cutis vera and the induration into the loose tissues beneath. When on a mucous membrane it involves its whole thickness and the induration is in the connective tissue beneath.

In any of its forms, the chancre may become phagedenic; then all the tissues, including the induration, die and slough out.

The induration may begin with the papule, or may be developed during the ulcerative process. Sometimes, however, it is only discovered after the healing of the chancre.

In a case of erosion or fissure, it only appears after they are healed, if at all.

The chancre has always a period of incubation—that is, a certain time elapses between the moment of inoculation and the appearance of the papule—this time varies considerably. It is rarely less than ten days, and is generally much longer. I have seen a case, that of a sailor, who was on board ship sixty-five days between Havre and this port. Two nights before leaving Havre, he cohabited with a prostitute, and discovered the "pimple," as he called it, only five days before reaching Savannah, so that the time of incubation in this case was sixty-two days.

The length of the period of incubation is of great importance in forming the diagnosis of chancre, for in any other inoculation, the parts will frequently present an inflamed appearance from the very first.

But the most reliable sign of syphilitic infection, in my opinion, is the enlargement of the lymphatic ganglia nearest to the point of inoculation. The poison spreads along the lymphatic vessels to these ganglia, and causes them to enlarge. They become hard, are movable, have a peculiar elastic feel to the touch, and are perfectly painless on pressure, unless inflamed. If the point of inoculation be on the face, mouth or throat, the ganglia of the neck will always be found affected, with perhaps those about the jaws or any other part of the head. If it be on the hand, those on the arm and in the axilla of the affected side; if on the genitals, the inguinal chain in both sides; if on the foot or leg or thigh, those on the limb and the groin.

In non-specific ulcers and in venereal ulcers, one gland usually, with sometimes the lymphatic vessel leading to it, becomes enlarged in one or both groins, but it is always inflamed, always painful, and usually suppurates in the end, when produced by a venereal ulcer. In the chancre, on the contrary, many glands will be found enlarged ; they will be small, painless and never suppurate except in broken down constitutions, or where the sore becomes inflamed by the treatment or some other cause.

Should the patient under your examination have a non-infectious excoriation, cut, fissure, pimple or wart upon the penis, there will be no enlargement of the *chain* of inguinal ganglia ; one or two of them may become tumefied, but this tumefaction will, under the application of water-dressing to the sore, disappear along with it, in a few days, never to return.

If the case be one of herpes, three or more watery pimples will be found in a cluster, at one or several different points, on the glans, the prepuce, or any other part of the penis. Herpes is seldom attended by any enlargement in the groin, unless irritating applications are used, and will under simple dressings disappear in five to seven days, but will frequently recur afterwards.

In syphilis the enlargement of the lymphatic ganglia may be so trifling that the most delicate manipulation only will detect it, and it may even be that a person not having much experience cannot detect it at all. In this case, as in all others in which *there is a doubt* as to diagnosis, the doctor must next resort to inoculation of the patient with the unmixed serum from the suspected sore, somewhat in the same manner that vaccination is made with the unmixed lymph.

If this inoculation produce *the like* of the original sore, and can be repeated with the same result, then the case is not syphilis.

But great care is required in getting only the pure virus on the point of the lancet,and in introducing it, so as not to cause unnecessary irritation, for the introduction of decaying tissue into the skin might be a source of danger in itself, and irritation might prevent the inoculation.

Where, in cases of erosion, fissue, etc., there is no reliable secretion to inoculate with, and the enlargement of the ganglia cannot be demonstrated with certainty, nothing is left but to wait and watch for the appearance of secondary symptoms.

A case of venereal ulcer presents a strikingly different appearance from the chancre or any other lesion. It first appears as a vesicle or pustule, inflamed all around and attended with burning and itching, in from one to five days after the coition which caused it. This rapidly changes into an open ulcer, irregular in form, with ragged, well-defined edges, forming a red, angry areola. (Sometimes the ulceration begins with a vesico-pustule). The surface of the ulcer is usually flat; its cavity contains yellow pus mixed with the gray disintegrating tissues. If some of the pus be taken on the point of a lancet and inserted under the epidermis, on any part of the patient's body, it will be followed in a few hours by an inflammation, and shortly afterwards by an ulcer of the same kind as the original, and this process may be repeated *ad infinitum* with the same results.

The venereal ulcer is frequently multiple; two or more may commence at the same time, or one after the other from self-inoculation. (If three or more appear in a cluster, they may be mistaken for herpes). They are always painful, and the patient is anxious for relief.

The ulcer has a tendency to spread, and, unless the virus be destroyed by early and deep cauterization, may get beyond control, even under the most careful treatment. It is almost always found on the genitals, but is sometimes seen in other situations, though not so frequently as chancre.

As the ulcerative process goes on upon the penis, the labia or contiguous parts, one, or rarely two of the ganglia in the superficial inguinal chain, on one or both sides, becomes inflamed, much enlarged and very painful.

The inflamed ganglion is always tender on pressure, and not movable as in syphilis; it increases rapidly in size, and this increase is often attended by a rise in the temperature and pulse, which may amount to a fever, continuing until suppuration takes place, and the pus evacuated.

Although the venereal ulcer may sometimes spread over a large portion of the body, it always remains a local disease. It is never constitutional, and mercury is contra-indicated.

Perhaps my country brother may be called upon to diagnose a case which presents all of the symptoms of a venereal ulcer, as above described; but let him beware of rashly telling his patient that he has no constitutional disease. For it sometimes happens that the virus of the chancre and that of the venereal ulcer have been inoculated together at the same spot. Then the venereal ulcer takes the lead, and progresses in its usual manner, but after ten days or more, the symptoms of syphilis can be discovered by close examination. Those ganglia of the inguinal chain, which have not been affected by the venereal ulcer, will be found enlarged, movable, painless, with the peculiar, hard, elastic feel before described. At the same time the button-like induration of a chancre will make its appearance under the ulcer, elevating it above the skin. If these symptoms escape notice, the doctor will be greatly mystified, at the end of six to ten weeks, by the discovery of a very significant eruption; or the patient may consult him for some trouble about the throat, when, upon examination, he may find one or more mucous patches staring him in the face.

Therefore, I repeat, be very guarded in expressing an opinion; be very slow in making a diagnosis.

Insisting again upon the necessity of examining the entire skin, and, as far as may be, the mucous membranes of the patient, in the search for a primary lesion, in every suspicious case, I now give the differential diagnosis between syphilis and venereal ulcer, because they are frequently mistaken the one for the other.

| THE CHANCRE. | THE VENEREAL ULCER. |
|---|---|
| 1. Is always the result of inoculation with virus from a chancre, or from one of the syphilo-dermata, or with the blood of a syphilitic. | 1. Is always the result of inoculation with the virus from a venereal ulcer or from its suppurating bubo. |
| 2. Has a long period of incubation, usually more than ten days. | 2. Has no period of incubation; may appear within twenty-four hours or after a few days. |
| 3. Is painless and will generally heal without treatment; gives rise to no fever. | 3. Is always painful, has a tendency to spread, requires careful treatment, gives rise to febrile excitement. |

| | |
|---|---|
| 4. Is generally single; if multiple, always so from the original inoculation. | 4. Is generally multiple from the beginning, or by fresh inoculations. |
| 5. Begins as a papule without an inflamed areola, or as an erosion. | 5. Begins as a pustule with an inflamed areola, or as an erosion. |
| 6. When fully developed, its form is that of a round or oval, cup-shaped ulcer, with adherent sloping edges. | 6 When fully developed, its form is that of an irregular, flat and deep ulcer, with overhanging, ragged edges, forming a red, angry areola. |
| 7. Its contents are serum, and the dead tissues gray and tough, like wet parchment, which will slough out without suppuration or inflammation. It has an indurated base, which elevates it above the surrounding skin, or mucous membrane. Induration button like in shape and feeling, painless, non-inflammatory and very persistent. The indurated chancre involves the whole thickness of the skin and of the areola tissues beneath. As an erosion, it has no definite shape, and may have no induration. | 7. Its contents, yellow pus, mixed with disintegrating gray tissues; has no induration at the base beyond the line of inflammation; rarely involves the tissues below the skin; is not elevated above the surrounding skin or mucous membrane. Beginning as an erosion, it soon takes the definite shape of the ulcer. |
| 8. Rarely becomes phagedenic; usually follows a regular course until cured. | 8. Has a tendency to phagedena; extensive ulcerations are relatively frequent; has an irregular course, and is stubborn to treatment. |
| 9. The virus can rarely be re-inoculated on the patient. | 9. Can always be re-inoculated on the patient any number of times. |
| 10. Always affects all the surrounding superficial, lymphatic ganglia. The enlargement usually trifling. These ganglia are hard, painless, freely movable, and do not suppurate. | 10. Never affects all the surrounding ganglia; one or two become inflamed, greatly enlarged and very painful. They generally suppurate; the pus often inoculable. |
| 11. Is always constitutional, *ab initio*. | 11. Always remains a local disease. |
| 12. When left to nature will heal, but after a length of time will be followed by other lesions upon the skin and mucous membranes. Later on every organ of the body may become affected by the disease. | 12. When left to nature the ulceration extends. It may spread over a large surface of the skin and become formidable, but once healed is not followed by any other manifestation. |
| 13. Mercury is the best remedy known to affect a cure. | 13. Mercury is useless, and often injurious in the treatment. |

### PROGNOSIS.

There is no single symptom, nor group of symptoms, in a case of primary or secondary syphilis, which will justify us in pronouncing a positive opinion as to the final result. It may be stated as a general rule, that when the primary symptoms are severe, the secondary manifestations will follow promptly, and be strongly marked, and when mild in its first stage the disease will be mild in the second.

But it must not be concluded that the mild case is more likely than the other to have a favorable termination. For, no matter how small the amount of the disease lingering in the system, it will certainly manifest itself in some way or other. Either the patient will be overtaken sometime during his life by so-called tertiary syphilis, or will be liable to complications in any disease by which he may be attacked, or to obscure diseases of the brain and nervous system, or remaining to all appearance healthy himself, his children may inherit constitutional syphilis.

Prominent syphilographers have held that the disease is "self-limited," and that it will disappear without treatment after an indefinite number of years, the patient being restored to perfect health. My own experience, however, is that in most cases where this appears to have occurred, the disease is not cured, but merely held in abeyance, to manifest itself when, either by the energy of its own poison, or by that of some other disease, the patient's vital force has been reduced.

I have seen numerous cases in which the patients left treatment before I thought them cured, and yet afterwards showed no symptoms, married, had healthy children and died of some other disease ; others, who, after several relapses and many doctors, have apparently gotten well after leaving all treatment.

On the other hand, cases, which had been treated and pronounced cured by eminent specialists in Europe and in America, have come under my observation at a later day with well-defined constitutional symptoms. It is then safe to assume that, without any treatment, the termination will be unfavorable ; that, under proper treatment, faithfully and persistently carried out, the disease will, in the great majority of cases, disappear in from eighteen to thirty-six months, never to return, leaving the patient in perfect health and not liable to infect his children.

There are, however, a small percentage of cases in which, even with the greatest skill on the part of the doctor, whether from neglect by the patient (which I believe is the most frequent cause), or from some peculiarity of constitution, skin manifestations, at times mild, at times inveterate, will return. In others, the patient, after remaining to all appearance perfectly well for years, will have his life ended by some obscure disease, which I consider as a result of syphilis.

I advise you, therefore, whenever called upon to give a prognosis in a case of syphilis, to do, as many years of experience and many disappointments have taught me to do—tell your patient that if he will faithfully carry out your directions, the chances of a cure are in his favor ; that the great majority of cases get well in this climate in about two years, under skillful treatment, but that you cannot promise a cure, because there are some cases which defy all treatment.

OUTLINE OF TREATMENT.

As it is now well understood that, during the incubation of the chancre, the syphilitic microbe travels along the lymphatic vessels into the ganglia, and at the time of the first appearance of the papule has already affected a lodgment in the system, it seems unnecessary for me to say, that all attempts to abort the disease by cauterization of the chancre will be ineffectual. Repeated experiments have shown that deep cauterization of the sore, even a few hours after its first appearance, fails to prevent constitutional symptoms.

The disease must be cured, if cured at all, by remedies which affect the general system.

It is desirable, of course, to heal the chancre as soon as possible, and to do this all irritant applications must be avoided. If, from any cause, there be irritation or inflammation around it, water-dressing, or weak astringent lotions, should be constantly kept applied to the parts, until it has disappeared, then a dry dressing of pulverized iodoform, clay, chalk or bismuth, etc., must be used night and morning, after carefully washing and drying the ulcer. By this treatment cleanliness is secured, the dead tissues will slough out readily, and the sore will then heal rapidly.

The enlarged ganglia require no treatment unless inflamed, still, it may be useful, in order to interest the patient in the treatment, or to relieve his apprehensions, to make him rub them over with some non-irritating ointment. But when the patient fully understands the dangers of neglect, it is always better to allow the absorption to be effected by the internal treatment, because the condition of the ganglia is a sure index of the progress of the cure.

The effect of the syphilitic microbe upon the human blood seems to be to cause the destruction of the red corpuscles. Almost every individual suffering from the first or second manifestations of syphilis, is anæmic. If the blood be examined under the microscope, it will be seen that the proportion of the large white corpuscle to the red, is much greater than in health.

Under the use of small doses of mercury and proper hygienic treatment, the red corpuscles increase rapidly, and the pale hue of the surface is replaced by a natural complexion.

The entire treatment then, of a case of syphilis, consists, according to my experience, in such hygienic measures as will bring the general health of the patient up to the highest standard possible, together with the administraion of that salt of mercury, which will increase the number of the red corpuscles.

The first duty of the physician, when the diagnosis of a case of syphilis is made, is to fully instruct his patient in all matters pertaining to the disease, warn him of its deplorable consequences, impress upon him the necessity of adhering rigidly to the treatment until a cure is effected, and get his promise to do so. But this promise alone avails nothing. I can recall many instances of young men of a class who would naturally be supposed to feel most fully the weight of a moral obligation, but who, while under such promise, have left the treatment as soon as the prominent symptoms had subsided, to die afterwards a miserable death from some of the sequelæ of the disease. After suffering many disappointments from this cause, the idea occurred to me of putting my patients under a money obligation to adhere to the treatment to the end, and I have found this plan to work very well as to results.

Next, a mode of life should be prescribed which will improve the patient's general health. Such diet only should be allowed as will build up the strength and not overtax the digestive powers. Alcoholic drinks and tobacco should be absolutely forbidden, as either of them will greatly interfere with the treatment.

Then the doctor must find, by repeated trials, that preparation of mercury which will improve the patient's blood, and the dose which he can take three times a day, for any length of time, without disturbing his digestion, and without inducing salivation, or tumefaction, or even reddening of the gums.

Individuals are met with who cannot take mercurials with advantage at first. Here we must alternate a tonic treatment with trials of the various salts of mercury until some one is found suitable to the case.

On the appearance of secondary symptoms, such non-irritant local application should be made to the affected parts as the judgment of the doctor may dictate, and the treatment previously pursued should be continued.

If constipation occur during the treatment, it should be relieved by a cathartic dose of calomel. When, after the disappearance of the secondary symptoms, the patient is restive under treatment, and irregular in taking his medicine, we may be obliged to produce an eruption of the skin, by the use of large doses of the iodides, in order to bring him under control.

It is never safe to discontinue the regular treatment in less than eighteen months after its commencement. If, at the end of that time, the patient has been free from all symptoms for six months, and the ganglia have returned to their normal size and condition, the mercury may be intermitted for longer and longer periods during the next six months, and then discontinued altogether, but the patient must be kept under observation for six months longer, when, if still free from symptoms, he may be discharged as cured.

It is always advisable to keep up the treatment for six months after the disappearance of the last symptoms, no matter how long the case may have lasted.

If the red corpuscles do not increase in number under mercury, it must be stopped for a while. If the general health does not improve, a change of air and climate must be tried.

Whenever the disease recurs after an apparent cure, the treatment indicated above must be resumed, as in a new case.

In cases where the deep tissues, or the internal organs, or the bones are involved, and swelling, or deposits are present, the iodides should be used to cause absorption, and stopped so soon as this result is reached.

It will be observed that my opinion of the value of the iodides, in the treatment of syphilis, differs from that usually entertained.

I have found that when their use is pushed to any extent, they impoverish the blood and impair the vital powers of the patient, while the theory of treatment on which I practice is, that all our efforts should be directed to building up.

In giving these simple directions, I think I have discharged the self-imposed duty of putting it in the power of a country physician to control any ordinary case of syphilis, and to conduct its treatment to a successful issue. For the details of treatment of the many complications which may arise during its progress, or which may otherwise come before him, I must refer him to works upon the general subject.

